# Supplementary figures and images for: Human mitochondrial carriers of the SLC25 family function as monomers exchanging substrates with a ping-pong kinetic mechanism
Source: EMBO J. 2024 Jun 27;43(16):3450–65. doi: 10.1038/s44318-024-00150-0 (PMC11329753; doi:10.1038/s44318-024-00150-0)

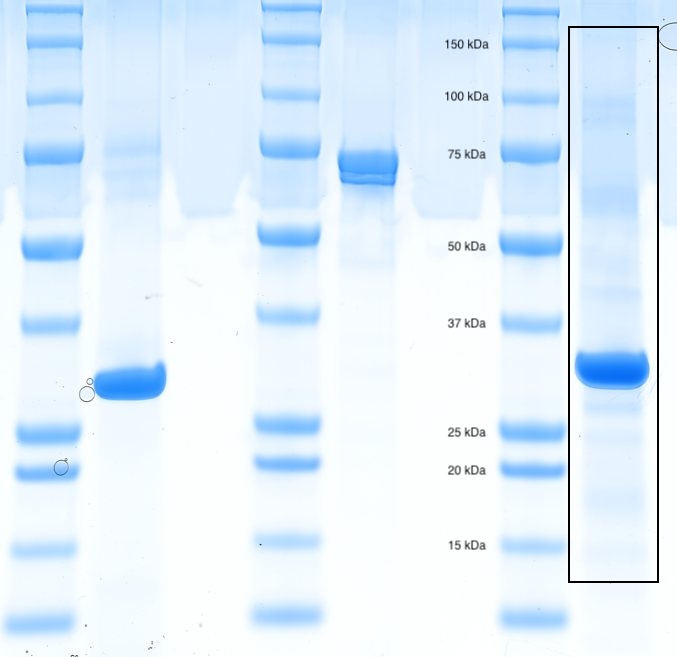

Supplement: Supplementary file 2 — Source data Fig. 1 [file 44318_2024_150_MOESM2_ESM.zip › Figure_1/Figure_1E/1E.jpg]

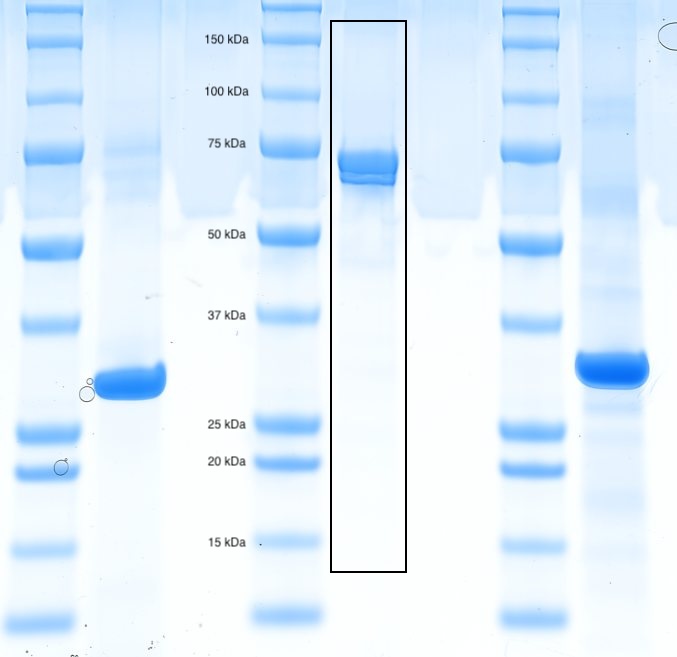

Supplement: Supplementary file 2 — Source data Fig. 1 [file 44318_2024_150_MOESM2_ESM.zip › Figure_1/Figure_1M/1M.jpg]

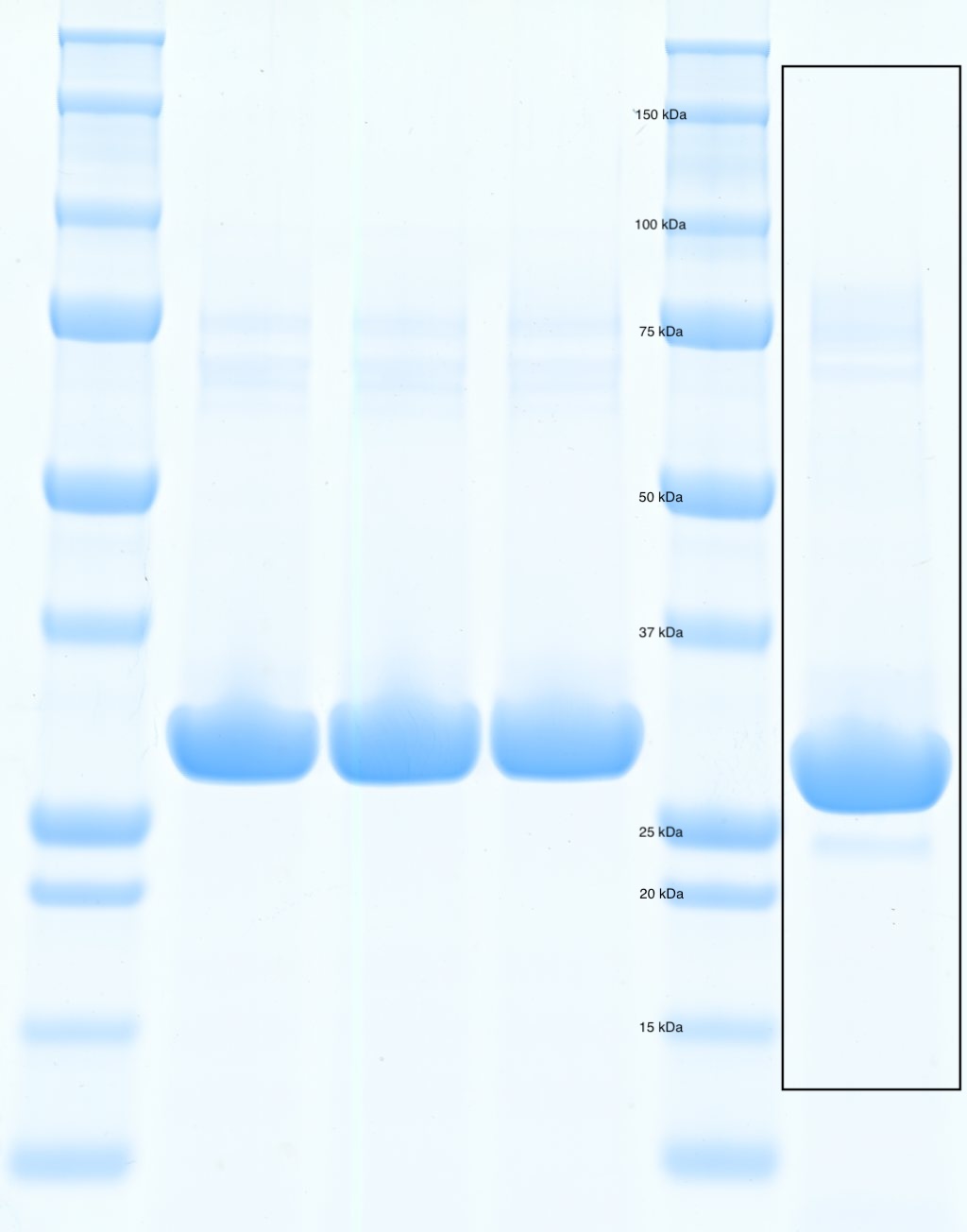

Supplement: Supplementary file 2 — Source data Fig. 1 [file 44318_2024_150_MOESM2_ESM.zip › Figure_1/Figure_1A/1A.jpg]

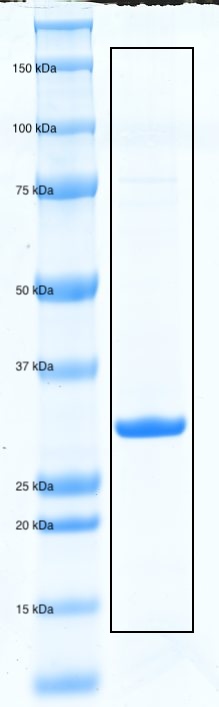

Supplement: Supplementary file 2 — Source data Fig. 1 [file 44318_2024_150_MOESM2_ESM.zip › Figure_1/Figure_1I/1I.jpg]
