# Supplementary material for: Human mitochondrial carriers of the SLC25 family function as monomers exchanging substrates with a ping-pong kinetic mechanism
Source: EMBO J. 2024 Jun 27;43(16):3450–65. doi: 10.1038/s44318-024-00150-0 (PMC11329753; doi:10.1038/s44318-024-00150-0)
Supplement: Supplementary file 6 — Expanded View Figures [file 44318_2024_150_MOESM6_ESM.pdf]

Expanded View Figures

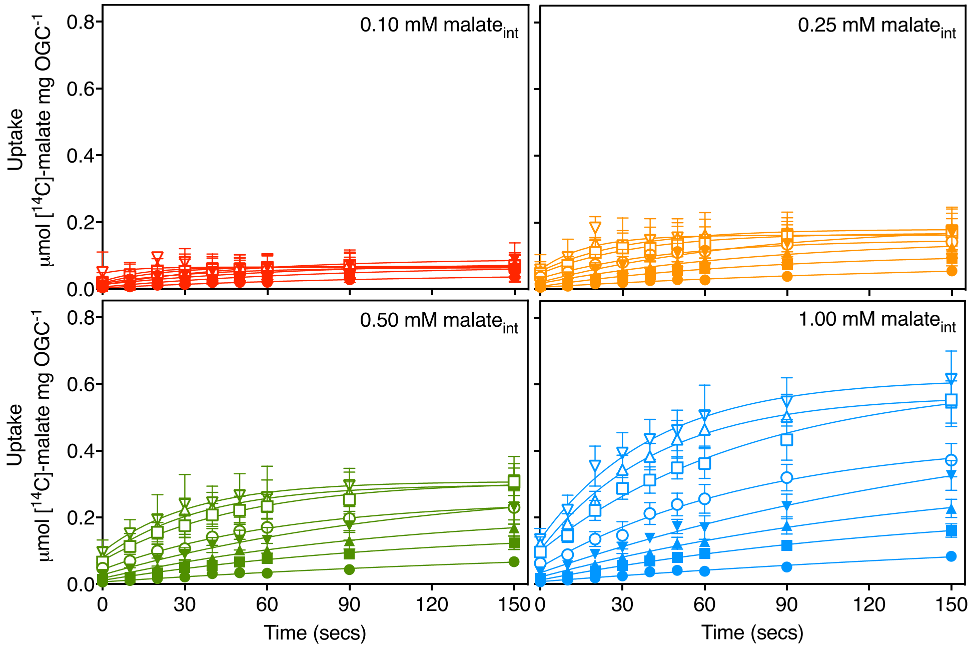

Figure EV1. Uptake of transport catalyzed by reconstituted human oxoglutarate carrier.

Proteoliposomes containing human oxoglutarate carrier were loaded with either 0.10 mM (red traces), 0.25 mM (orange traces), 0.50 mM (green traces), or 1.00 mM (blue traces) malate$_{int}$, and the transport was initiated by the externally added radiolabeled malate at either 0.5 µM (filled circles), 1.0 µM (filled squares), 1.5 µM (filled upward triangles), 2.5 µM (filled downward triangles), 5.0 µM (open circles), 10 µM (open squares), 15 µM (open upward triangles), or 20 µM (open downward triangles) [^{14}C]-malate (malate$_{ext}$). Initial rates were estimated by fitting the uptake data to Eq. 1. The data represent the average and standard deviation of $n = 6$ (two independent experiments, each with three technical repeats). Source data are available online for this figure.

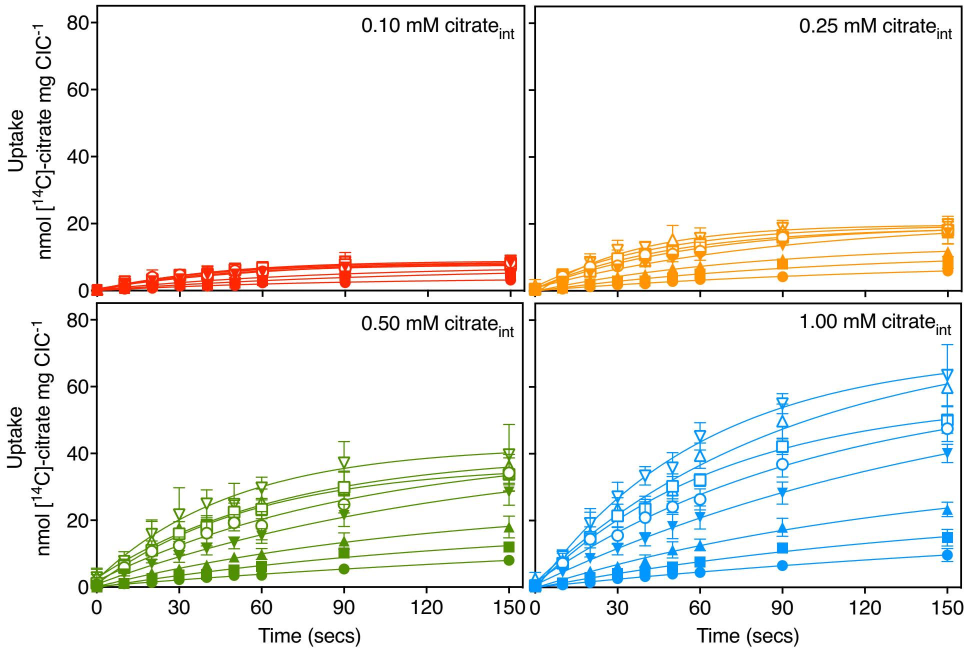

Figure EV2. Uptake curves of the transport catalyzed by reconstituted human citrate carrier.

Proteoliposomes containing human citrate carrier were loaded with either 0.10 mM (red traces), 0.25 mM (orange traces), 0.50 mM (green traces), or 1.00 mM (blue traces) citrate$_{int}$, and transport was initiated by the externally added radiolabeled sulfate at either 1.0 μM (filled circles), 1.5 μM (filled squares), 2.5 μM (filled upward triangles), 5 μM (filled downward triangles), 10 μM (open circles), 15 μM (open squares), 20 μM (open upward triangles), or 25 μM (open downward triangles) [^{14}C]-citrate (citrate$_{ext}$). Initial rates were estimated by fitting the uptake data to Eq. 1. The data represent the average and standard deviation of $n = 6$ (two independent experiments, each with three technical repeats). Source data are available online for this figure.

 © The Author(s)

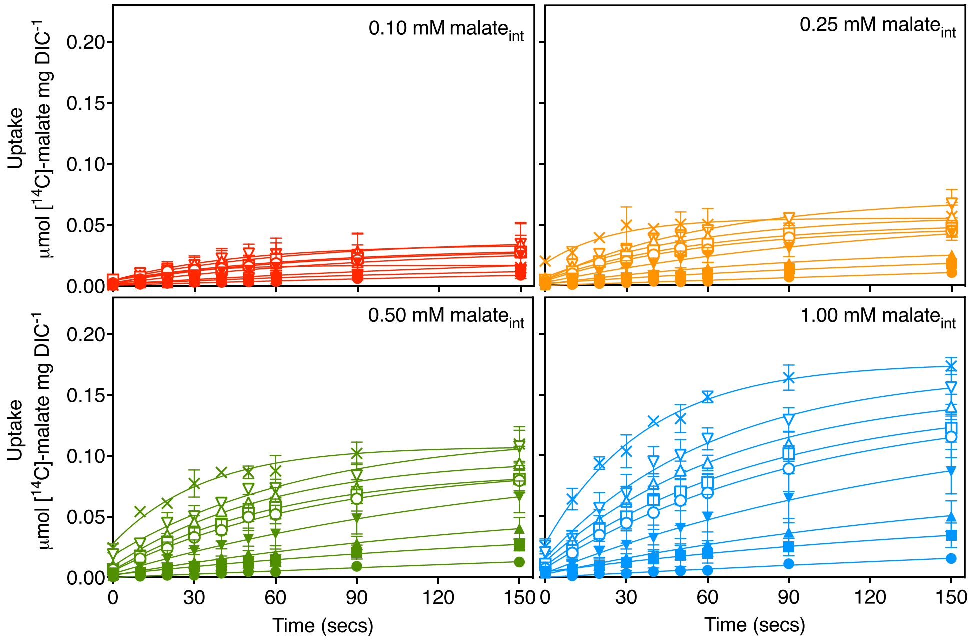

Figure EV3. Uptake curves of the transport catalyzed by reconstituted human dicarboxylate carrier.

Proteoliposomes containing human dicarboxylate carrier were loaded with either 0.10 mM (red traces), 0.25 mM (orange traces), 0.50 mM (green traces), or 1.00 mM (blue traces) malate$_{int}$, and transport was initiated by the externally added radiolabeled malate at either 1.0 µM (filled circles), 1.5 µM (filled squares), 2.5 µM (filled upward triangles), 5 µM (filled downward triangles), 10 µM (open circles), 15 µM (open squares), 20 µM (open upward triangles), 25 µM (open downward triangles), or 50 µM (crosses) [^{14}C]-malate (malate$_{ext}$). Initial rates were estimated by fitting the uptake data to Eq. 1. The data represent the average and standard deviation of $n = 6$ (two independent experiments, each with three technical repeats, except the 1 and 50 µM external [^{14}C]-malate datasets, which are the average of three technical repeats). Source data are available online for this figure.

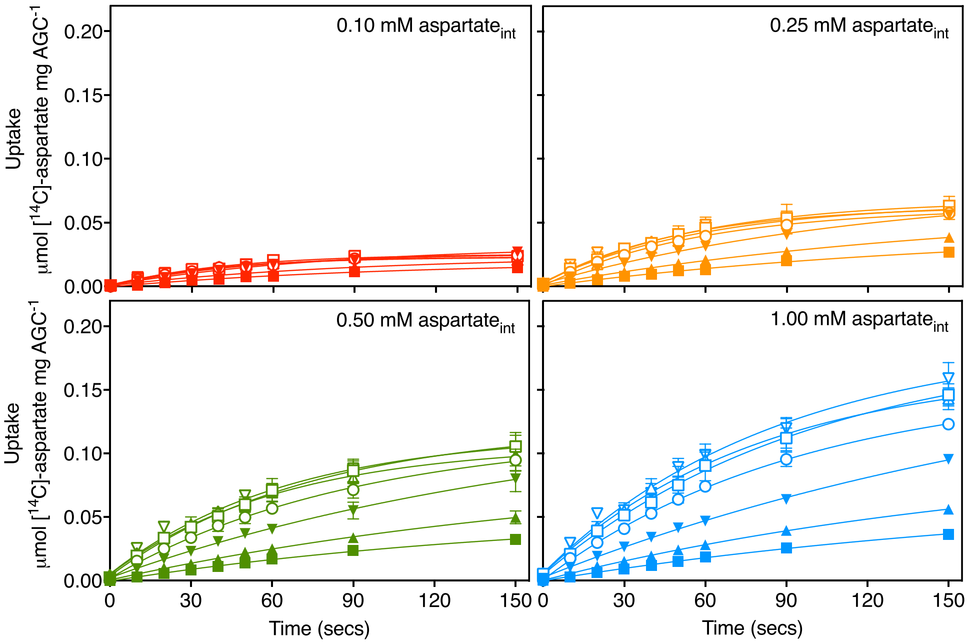

Figure EV4. Uptake curves of the transport catalyzed by reconstituted human aspartate/glutamate carrier.

Proteoliposomes containing human aspartate/glutamate carrier were loaded with either 0.10 mM (red traces), 0.25 mM (orange traces), 0.50 mM (green traces), or 1.00 mM (blue traces) aspartate$_{int}$, and the transport was initiated by the externally added radiolabeled aspartate at either 1.5 µM (filled squares), 2.5 µM (filled upward triangles), 5.0 µM (filled downward triangles), 10 µM (open circles), 15 µM (open squares), 20 µM (open upward triangles), or 25 µM (open downward triangles) [^{14}C]-aspartate (aspartate$_{ext}$). Initial rates were estimated by fitting the uptake data to Eq. 1. The data represent the average and standard deviation of three technical repeats. Source data are available online for this figure.

 © The Author(s)
